# Supplementary material for: Explaining Echis: Proteotranscriptomic Profiling of Echis carinatus carinatus Venom
Source: Toxins (Basel). 2025 Jul 16;17(7):353. doi: 10.3390/toxins17070353 (PMC12298760; doi:10.3390/toxins17070353)
Supplement: Supplementary file 1 [file toxins-17-00353-s001.zip › Supplementary_Figures_Revised.pdf]

Supplementary Figure S1A: The RP-HPLC of *E. c. carinatus* EcCaKA08 venom.

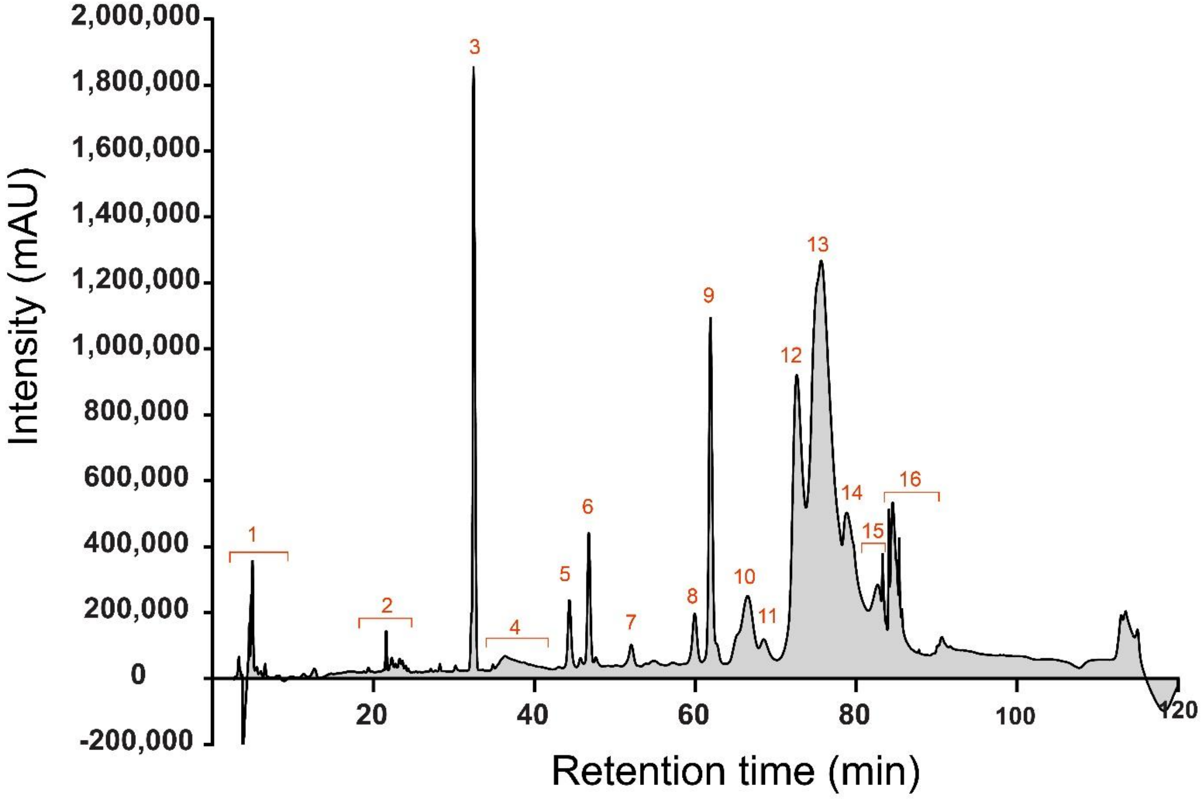

The RP-HPLC profile of *E. c. carinatus* is illustrated here. The distinct peaks selected for mass-spectrometry analysis are numbered in red colour.

Supplementary Figure S1B: The SDS_PAGE of *E. c. carinatus* EcCaKA08 venom after HPLC.

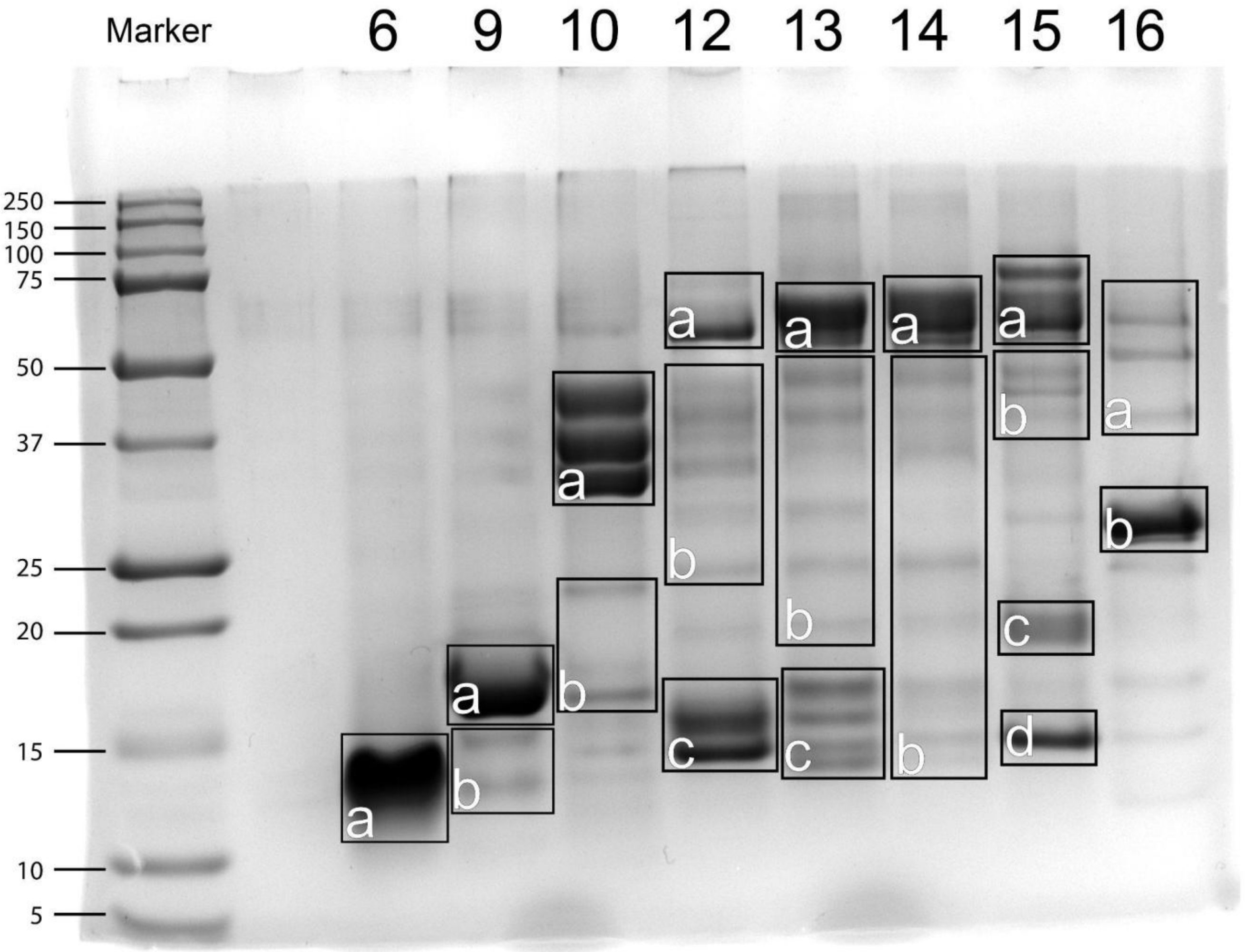

The SDS-PAGE for the eight major peaks of *E. c. carinatus* venom after RP-HPLC is depicted here. Each lane was excised as shown above (marked as a, b, c, or d) and subjected to in-gel digestion before mass spectrometry.
