# Supplementary material for: Explaining Echis: Proteotranscriptomic Profiling of Echis carinatus carinatus Venom
Source: Toxins (Basel). 2025 Jul 16;17(7):353. doi: 10.3390/toxins17070353 (PMC12298760; doi:10.3390/toxins17070353)
Supplement: Supplementary file 1 [file toxins-17-00353-s001.zip › Supplementary_Tables_revised.pdf]

Supplementary Table S1A: The number of reads generated in the transcriptome after trimming the adapters.

Sample	Generated reads	Surviving reads	Percentage surviving reads
EcCaKA22_VGR	36,216,346	34,573,299	95.46%
EcCaKA22_VGL	30,135,873	28,360,449	94.11%
EcCaKA22_M	80,757,205	78,695,370	97.45%
EcCaKA05_VGR	52,266,080	51,198,196	97.96%
EcCaKA05_VGL	50,956,821	49,964.181	98.05%
EcCaKA05_Int	64,422,162	62,996,435	97.79%

Supplementary Table S1B: RNA-sequencing *de novo* assembly results and BUSCO group percentages after completeness analysis.

Sample	Total transcripts	Percent GC content	N50	BUSCO		
				Complete gene representation	Partially recovered	Missing
EcCaKA22	735,540	41.76%	580	83.13%	4.47%	12.41%
EcCaKA05	587,908	42.82%	1,424	84.92%	2.74%	12.34%

Supplementary Table S1C: Transcript level quantification and alignment.

Sample	Overall alignment rate	Not aligned
EcCaKA22_VGR	85.46%	14.54%
EcCaKA22_VGL	85.71%	15.29%
EcCaKA22_M	86.18%	13.82%
EcCaKA05_VGR	83.50%	16.50%
EcCaKA05_VGL	83.27%	16.73%
EcCaKA05_Int	87.11%	12.89%

Supplementary Table S2: Normalisation of Mass-spectrometry quantification using RP-HPLC and SDS-PAGE.

Peak fraction no	HPLC peak area	Densitometry	Relative peak fraction	Digestion type
F1	0.00728	1	0.00728	In-Solution
F2	0.00857	1	0.00857	In-Solution
F3	0.06521	1	0.06521	In-Solution
F4	0.01589	1	0.01589	In-Solution
F5	0.01117	1	0.01117	In-Solution
F6a	0.02204	1	0.02204	In-Gel
F7	0.00514	1	0.00514	In-Solution
F8	0.01193	1	0.01193	In-Solution
F9a	0.06056	0.719614854	0.043579876	In-Gel
F9b	0.06056	0.280385146	0.016980124	In-Gel
F10a	0.04231	0.74016196	0.031316253	In-Gel
F10b	0.04231	0.25983804	0.010993747	In-Solution
F11	0.0087	1	0.0087	In-Solution
F12a	0.14203	0.155685657	0.022112034	In-Gel
F12b	0.14203	0.47954702	0.068110063	In-Gel
F12c	0.14203	0.364767323	0.051807903	In-Gel
F13a	0.39019	0.265215193	0.103484316	In-Gel
F13b	0.39019	0.448725986	0.175088392	In-Gel
F13c	0.39019	0.286058821	0.111617291	In-Gel

F14a	0.10199	0.391166715	0.039895093	In-Gel
F14b	0.10199	0.608833285	0.062094907	In-Gel
F15a	0.04458	0.41255196	0.018391566	In-Gel
F15b	0.04458	0.176001273	0.007846137	In-Gel
F15c	0.04458	0.21944398	0.009782813	In-Gel
F15d	0.04458	0.192002787	0.008559484	In-Gel
F16a	0.06241	0.509227317	0.031780877	In-Gel
F16b	0.06241	0.490772683	0.030629123	In-Gel

Supplementary Table S3. The median lethal dose of *E. c. carinatus* venom.

Name of sample	Venom dose (μg)					Number of survivors					LD_{50} (μg/mouse)	LD_{50} (mg/kg)
EcCaKA (pooled)	8.7	11.4	14.8	19.2	25	3	4	2	0	0	15.06<br>10.3-22.02	0.753<br>0.515-1.101

Supplementary Table S4. Details of antivenom samples investigated in this study.

**Manufacturer**	**Batch**	**Manufacture (M) and expiry (E) dates**	**Marketed neutralising potency**
Bharat Serums and Vaccines Ltd.	A05318020	M: 01/2018<br>E: 12/2021	*Echis carinatus*: 0.45 mg/ml
Haffkine BioPharmaceutical Corporation Ltd.	AS101003	M: 10/2010<br>E: 03/2015	
Premium Serums & Vaccines Pvt. Ltd.	012014	M: 11/2014<br>E: 10/2018	
VINS Bioproducts Ltd.	01AS23049	M: 04/2023<br>E: 03/2027	
Biological E Ltd.	BAS00218	M: 02/2018<br>E: 01/2022	
